# Dapper Antagonist of Catenin-1 Cooperates with Dishevelled-1 during Postsynaptic Development in Mouse Forebrain GABAergic Interneurons

**DOI:** 10.1371/journal.pone.0067679

**Published:** 2013-06-24

**Authors:** Annie Arguello, XiaoYong Yang, Daniel Vogt, Amelia Stanco, John L. R. Rubenstein, Benjamin N. R. Cheyette

**Affiliations:** 1 Biomedical Sciences Graduate Program, University of California San Francisco, San Francisco, California, United States of America; 2 Department of Psychiatry, Nina Ireland Laboratory of Developmental Neurobiology, University of California San Francisco, San Francisco, California, United States of America; Virginia Tech Carilion Research Institute, United States of America

## Abstract

Synaptogenesis has been extensively studied along with dendritic spine development in glutamatergic pyramidal neurons, however synapse development in cortical interneurons, which are largely aspiny, is comparatively less well understood. Dact1, one of 3 paralogous Dact (Dapper/Frodo) family members in mammals, is a scaffold protein implicated in both the Wnt/β-catenin and the Wnt/Planar Cell Polarity pathways. We show here that Dact1 is expressed in immature cortical interneurons. Although Dact1 is first expressed in interneuron precursors during proliferative and migratory stages, constitutive *Dact1* mutant mice have no major defects in numbers or migration of these neurons. However, cultured cortical interneurons derived from these mice have reduced numbers of excitatory synapses on their dendrites. We selectively eliminated Dact1 from mouse cortical interneurons using a conditional knock-out strategy with a Dlx-I12b enhancer-Cre allele, and thereby demonstrate a cell-autonomous role for Dact1 during postsynaptic development. Confirming this cell-autonomous role, we show that synapse numbers in Dact1 deficient cortical interneurons are rescued by virally-mediated re-expression of Dact1 specifically targeted to these cells. Synapse numbers in these neurons are also rescued by similarly targeted expression of the Dact1 binding partner Dishevelled-1, and partially rescued by expression of Disrupted in Schizophrenia-1, a synaptic protein genetically implicated in susceptibility to several major mental illnesses. In sum, our results support a novel cell-autonomous postsynaptic role for Dact1, in cooperation with Dishevelled-1 and possibly Disrupted in Schizophrenia-1, in the formation of synapses on cortical interneuron dendrites.

## Introduction

Cortical function requires a balance between excitatory and inhibitory neurotransmission. Imbalance between excitatory and inhibitory neurotransmission can lead to epilepsy [1], impaired cognition [2], and is theorized to underlie other neuropsychiatric conditions [3], [4], [5]. Cortical presynaptic excitation is mediated by glutamatergic projection neurons, typically pyramidal in morphology with spiny dendrites, whereas presynaptic inhibition is mediated by various subtypes of GABAergic interneurons that generally have smooth (aspiny) dendrites [6]. The formation of synapses and dendritic spines has been a subject of much study in pyramidal neurons. In comparison, the formation of synapses on the aspiny dendritic shafts of interneurons has been less fully characterized and is less well understood [7], [8], [9].

Multiple molecular mechanisms control synapse development [10], including Wnt signaling, which encompasses a set of molecularly overlapping intercellular communication pathways [11], [12]. The major subdivisions of Wnt signaling, the β-catenin-dependent, Planar Cell Polarity (PCP), and Ca^2+^ pathways, have all been implicated in synapse formation through the organization of presynaptic sites at axon terminals [13], [14], [15], [16] and at postsynaptic sites along dendrites [17], [18], [19], [20]. Dact1 is an intracellular scaffold protein implicated in both the Wnt/β-catenin and Wnt/PCP pathways [21], [22], [23], [24], [25]. *Dact1* is expressed in the developing and adult mouse forebrain [26] and is required within pyramidal neurons for normal spine and excitatory synapse formation [11]. *Dact1* gene expression is also upregulated in subpallial-derived GABAergic interneurons during their migration into the developing cortex [27], [28]. We show here that during embryonic development, *Dact1* is expressed in interneuron progenitors of the ventral telencephalon as well as their derivatives in the cortical plate. Although Dact1 is expressed in migratory immature interneurons, *Dact1* null mutant mice show no obvious defects in the migration, distribution, or numbers of these interneurons in the developing cortex. However, these mice do have defects in the number of synapses on cortical interneuron dendrites. Using a conditional knock out strategy, we show that these neurodevelopmental phenotypes reflect a cell autonomous postsynaptic requirement for Dact1 in interneurons. We further show that interneuron-specific expression of Dact1, its binding partner Dishevelled-1 (Dvl1) [21], or Disrupted in Schizophrenia-1 (DISC1), a gene implicated in psychiatric pathogenesis, all significantly rescue synapse numbers in Dact1-deficient interneurons.

The results presented here demonstrate a novel cell-autonomous postsynaptic role for Dact1 in cortical interneurons. On the basis of these studies, we propose that Dact1 and Dvl1, acting in conjunction with or in parallel to DISC1, cooperate in the assembly and maintenance of the postsynaptic compartment in cortical interneurons.

## Materials and Methods

### Ethics Statement

All experimental procedures were carried out in accordance with the National Institutes of Health guidelines for the ethical treatment of animals. The Institutional Animal Care and Use Committee (IACUC) at the University of California San Francisco approved the animal protocol for this study (Protocol Number: AN084465-02A). All mice were deeply anesthetized and decapitated prior to brain tissue removal and all efforts were made to minimize suffering.

### Animals

*Dact1* alleles, as described in the Results, are all derived from the *Dact1^tm1.Bnrc^* targeted allele generated in the Cheyette lab [25]. *Lhx6-GFP*, a bacterial artificial chromosome (BAC) transgenic line, was obtained from The Gene Expression Nervous System Atlas (GENSAT) Project at Rockefeller University (New York, NY). Tg(I12b-Cre)1Jlr (*I12bCre*), a transgenic mouse line in which the Cre recombinase is under the control of an ultra-conserved DNA element near the *Dlx1* and *Dlx2* locus, was previously described [29], as was Tg(CAG-cat-EGFP)39Miya (CAG-cat-EGFP), a transgenic line that expresses GFP upon Cre mediated recombination [30], and *Gad1^tm1.1Tama^* [31], a glutamic acid decarboxylase-green fluorescence protein knock-in transgenic mouse line. Constitutive *Dact1 KO*'s and controls were littermate offspring of a *Dact1^−/+^;Lhx6*-GFP^/+^ intercross. Mice lacking Dact1 selectively in tangentially migrating GABAergic neurons, i.e. Interneuron-specific *Dact1 KO*'s (*Dact1^flox/flox^;I12bCre/CAG-cat-eGFP*) and controls, were littermate offspring of a male *Dact1^flox/flox^;I12bCre* and female *Dact1^flox/+^;CAG-cat-eGFP* intercross.

### Histology

Pregnant females were euthanized with carbon dioxide followed by cervical dislocation. E14.5 and E18.5 pups were extracted from the uterus and brains dissected and fixed with 4% paraformaldehyde (PFA) in phosphate buffered saline (PBS). Postnatal day 30 (P30) mice were deeply anesthetized with Avertin (Sigma) and intracardially perfused with PBS followed by 4% PFA. Brains were removed and post-fixed overnight in 4% PFA at 4°C, followed by cryoprotection by immersion in 30% sucrose in PBS at 4°C overnight. Embryonic brains were frozen in equal parts 30% sucrose and OCT (Tissue-Tek) and P30 brains in 100% OCT, on dry ice and stored at −80°C. Brains were cut at 20 µm on a Leica cryostat and mounted on Tissue Path Superfrost/Plus gold (Fisher Scientific) slides.

### Antibodies

Primary antibodies used include: rabbit anti-GFP and mouse anti-GFP (Invitrogen), chicken anti-GFP (Aves), rabbit anti-vesicular GABA transporter (VGAT), mouse anti-gephyrin, and rabbit anti-vesicular glutamate transporter 1 (VGLUT1) (Synaptic Systems), mouse anti-postsynaptic density-95 (PSD95) (NeuroMAB), rabbit anti-PSD95 (#3409 Cell Signaling), rabbit anti-DsRed (Clontech), mouse anti-HA (Cell Signaling), and rat anti-RFP (ChromoTek). Rabbit anti-Dact1 was previously described [11], rat anti-HA (Roche), mouse anti-FLAG (Sigma), and mouse anti-β-actin (Santa Cruz Biotechnology) were also used.

Secondary antibodies for immunoblot were HRP-conjugated (Thermo Fisher Scientific). Fluorescent secondary antibodies for immunofluorescence were anti-rabbit, anti-mouse, or anti-chicken Alexa Fluor 488-, 568-, or 647- conjugated (Invitrogen).

### Primary neuronal cultures

Cortical neuronal cultures were prepared from postnatal day 0 (P0) mouse brains as previously described [32]. High density cultures were plated at 1.25×10^5^ cells per cm^2^ on cover slips (12 mm; Fisher Scientific) previously coated with poly-L-lysine (10 µg/µl; Sigma) followed by laminin (5 µg/µl; Invitrogen) in a 2 cm^2^, 24 well plate.

### Plasmid construction and transfection

We constructed a lentiviral vector with the Dlx-I12b interneuron specific promoter [29] driving mCherry 5′ to the Thosea asigna virus (T2A) peptide and a multiple cloning site (MCS). Briefly, the Dlx-I12b enhancer and a beta-globin minimal promoter were subcloned upstream of an mCherry coding domain and then the Dlx-I12b enhancer, minimal promoter and mCherry gene were digested with 5′ BamHI and 3′ BsrGI and cloned into a lentiviral vector upstream of a T2a sequence and an MCS to generate the final vector. Next, we individually sub-cloned several cDNAs into the SphI site of the MCS (in frame to the T2a sequence) of this lentiviral construct for efficient infection and specific expression in interneurons: mouse Dact1, Rac1-CA (G12V; University of Missouri-Rolla cDNA Resource Center), HA-tagged mouse Dvl1, or HA-tagged mouse DISC1. To test for successful subcloning and recombinant expression of targeted proteins, Human Embryonic Kidney (HEK) 293T cells were transfected with each lentiviral construct using Fugene6 (Promega). HEK293T cells were maintained in Dulbecco's modified Eagle's medium (Invitrogen) supplemented with 10% heat-inactivated fetal bovine serum and 1% penicillin/streptomycin. Transfected cells were detected by expression of mCherry. At 3 days post-transfection, at which >80% of cells expressed mCherry, cells were collected and standard immunoblotting performed on the target protein to confirm successful expression of the intended subcloned cDNA. All oligonucleotides for PCR were synthesized by Integrated DNA Technologies services and all restriction enzymes were from New England Biolabs.

### Viral production and infection

Lentivirus was produced by co-transfection of lentiviral plasmids containing genes of interest and the helper plasmids pVSVG, pRRE, and pRSV into HEK293T cells using Fugene6 (Promega). 4 hours after transfection media was changed and 48 hours after transfection cells were checked for mCherry expression to determine transfection efficiency. At 4 days post transfection, at which >80% of cells were expressing mCherry, media was collected, centrifuged at 1200 rpms to remove debris/cells, and filtered through a 0.45 µm syringe filter. Unconcentrated virus was aliquoted and stored at −80°C.

For infection of 10 days *in vitro* (DIV) cortical neuronal cultures, lentivirus solution was warmed briefly in a 37°C water bath and then added directly, at a dilution of 1∶10, to 24 well plates. Media was changed 24 hours after infection followed by incubation until cells were fixed with 4% PFA at 15DIV.

### In situ hybridization

*In situ* RNA hybridization experiments were performed using digoxigenin-labeled RNA riboprobes on 20 µm frozen sections as previously described [33]. Riboprobes used included Dact1 [26], Lhx6 and Dlx1 [33].

### Immunofluorescence staining

#### Immunocytochemistry

Cells were fixed for 15 minutes with 4% PFA in PBS. After fixation, cells were washed in PBS and incubated for 1 hour in blocking solution (10% bovine serum albumin (BSA), and 0.3% Triton X-100 in PBS), followed by incubation in primary antibody diluted in blocking solution overnight at 4°C. After three 5 minute washes in PBT (0.3% Triton X-100 in PBS) cells were incubated in secondary antibodies diluted in blocking solution for 2 hours at room temperature. After three more 5 minute washes in PBT, cells were washed a final time in PBS and cover slips mounted on slides in Mowiol (Thermo Fisher Scientific).

#### Immunohistochemistry

P30 mounted brain sections were pre-warmed in PBS for 10 minutes at 37°C. Sections triple labeled for chicken GFP (1∶400; Aves)/mouse VGLUT1 (1∶200; Synaptic Systems)/rabbit PSD95 (1∶200; Cell Signaling) were treated with 4 mg/ml pepsin (DAKO) for 10 minutes at 37°C. Sections triple labeled for chicken GFP (1∶400; Aves)/rabbit VGAT (1∶400)/mouse Gephyrin (1∶200; Synaptic Systems) were treated with 1 mg/ml pepsin for 10 minutes at 37°C. Sections were then briefly rinsed in PBS, followed by PBT (0.3% Triton X-100 in PBS) and incubated in blocking solution (1% goat serum and 5% BSA in PBT) for 30 minutes at room temperature. Slides were placed in a humid chamber and incubated in primary antibodies at 4°C overnight for either one (ChkGFP/RbVGAT/MsGephyrin) or two (ChkGFP/MsVGLUT1/RbPSD95) days in blocking solution. After three 5 minute washes in PBT, sections were incubated in secondary antibodies diluted in blocking solution for 2 hours at room temperature. Sections were then washed in PBT, three times for 5 minutes, followed by a brief wash in PBS, and cover-slipped using Mowiol.

### Immunoblotting and co-Immunoprecipitation

As previously described [34].

### Image Analysis

Fluorescence images were acquired on a Nikon Spectral C1si confocal or Nikon Spinning Disk Confocal using a 40×oil, 60×oil, or 100×oil objective. All images were analyzed using NIH ImageJ software. Synaptic puncta quantification was performed on high density primary cortical cultures as described [35]. Only synaptic puncta with an area of 0.1 µm^2^–5 µm^2^ were counted along multiple GFP^+^ labeled primary dendrites of an individual neuron, from the cell soma to its first branch point. 40–60 neurons total were analyzed for each genotype and each condition, collected from multiple different wells, and at least 3 animals per genotype.

GFP^+^ interneurons in upper layers of the primary somatosensory cortex were selected for synaptic puncta co-localization in P30 brain sections. Co-localization was scored if pre- and post-synaptic puncta along GFP^+^ labeled primary dendrites overlapped by at least 1 pixel [36]. Co-localized puncta visible in adjacent serial sections were scored only once. 20–30 neurons total were analyzed for each condition and each genotype, collected from at least 3 animals per genotype.

### Statistics

Prism software (Graphpad) was used for data analysis and graph generation. All p-values were calculated by unpaired, parametric, two-tailed t-test (comparisons between two groups) or one-way ANOVA (comparisons among three or more groups) followed by Dunnetts test. Error bars indicate s.e.m.

## Results

### *Dact1* is expressed in Dlx-dependent interneuron precursors and immature cortical interneurons

**Figure 1:**
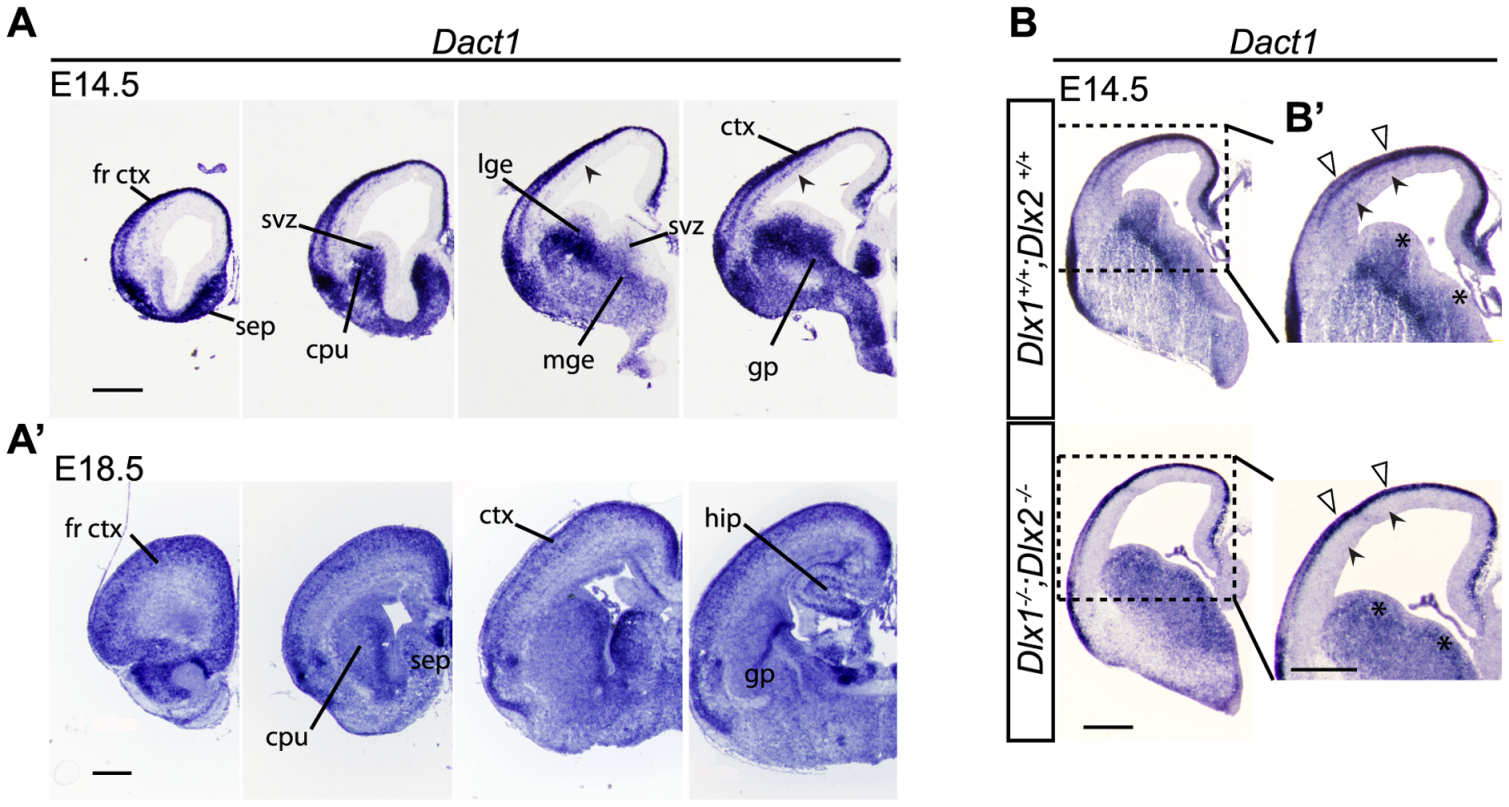
*Dact1* expression in the developing forebrain. *In situ* hybridization (Dact1) in rostral to caudal coronal sections through wild type E14.5 (**A**) and E18.5 brains (**A**'). Arrowheads in **A** indicate *Dact1* expression in the deep migratory stream (subventricular zone/intermediate zone) of ganglionic eminence-derived interneurons within the developing cortex. *In situ* hybridization (Dact1) at E14.5. **B** *Dact1* expression in *Dlx1^−/−^;Dlx2^−/−^* mice (bottom) compared to wild type (top) and higher magnification (**B**'), shows the absence (closed arrowheads) of *Dact1* expression in *Dlx1^−/−^;Dlx2^−/−^* mutants in the usual position of the deep migratory stream, indicating that Dact1 is normally expressed in the immature interneurons that fail to migrate in this *Dlx* double mutant. Asterisks in **B**' indicate the shift in Dact1 mRNA expression from the subventricular zone to the ventricular zone of the ganglionic eminences in *Dlx1^−/−^;Dlx2^−/−^* mice. *Dact1* remains expressed in the marginal zone of the cortex in the double mutants (open arrowheads), reflecting Dlx-independent expression in developing excitatory neurons. cpu, caudate-putamen; ctx, cortex; fr ctx, frontal cortex; gp, globus pallidus; hip, hippocampus; sep, septum; svz, subventricular zone. Scale bars = 500 µm.

*Dact1* has previously been reported to be expressed in the developing central nervous system (CNS), as well as in several regions of the mature CNS including the cerebellum, cortex, and hippocampus [26], [27], [33]. Here we focused on expression in developing cortical interneurons and their precursors. *In situ hybridization* (ISH) at embryonic day 14.5 (E14.5) showed *Dact1* expression in the telencephalic ganglionic eminences (GE), as well as their basal ganglia derivatives, caudate-putamen (lateral ganglionic eminence; LGE) and globus pallidus (medial ganglionic eminence; MGE). Dact1 mRNA was also expressed in the marginal zone, subplate, and subventricular zone of the developing cortex (Figure 1A). The expression pattern of *Dact1* in the E14.5 cortex is reminiscent of the distribution of tangentially migrating immature interneurons that are derived from the GE's (Figure 1A; as indicated by arrowheads). This is consistent with previous findings that *Dlx1* and *Dlx2* repress mRNA expression of *Dact1* in the ganglionic eminences [33] and that *Dact1* gene expression increases in GE-derived interneurons during their migration to the cortex between E13.5 and E15.5 [27], [28]. Nonetheless, Dact1 continues to be expressed at E18.5, far beyond the developmental peak in tangential migration of immature interneurons. ISH at E18.5 revealed substantial *Dact1* expression in the caudate-putamen, globus pallidus, hippocampus, as well as in neurons distributed across multiple layers of the developing cortical plate (Figure 1A').

The DLX family of homeobox transcription factors play an important role in the maturation, migration, and survival of GABAergic neurons [32], [37], [38], [39], [40]. In *Dlx1* and *Dlx2* double mutant mice, a large majority of GE-derived interneurons fail to migrate to the cortex, hippocampus, and olfactory bulbs, resulting in the accumulation of these cells in the GE [37], [41], [42]. Interestingly, in *Dlx1^−/−^;Dlx2^−/−^* mice, Dact1 mRNA expression is also altered (Figure 1B). There is an increase of *Dact1* expressing cells in the ventricular zones of the GE's (Figure 1B'; as indicated by asterisks) and an absence of *Dact1* expression in the region where immature interneurons migrate along their deep migration pathway to the cortex (Figure 1B'; as indicated by arrowheads). Thus, *Dact1* is expressed in interneuron precursors in the GE's and in immature interneurons that migrate tangentially from the GE's to the cortex. In *Dlx1^−/−^;Dlx2^−/−^* mutant mice, these Dact1-expressing immature interneurons fail to reach the cortex, however *Dact1* is still expressed in immature glutamatergic neurons of the cortical plate (Figure 1B'; as indicated by open arrowheads), consistent with our prior work demonstrating that Dact1 regulates development in forebrain pyramidal neurons [11].

### Constitutive *Dact1* mutant mice have no major defects in the production or tangential migration of GE-derived cortical interneurons

The expression of *Dact1* in immature cortical interneurons at proliferative and migratory stages suggests that *Dact1* could regulate production or migration of these cells. We hypothesized that the pattern of tangentially migrating interneurons would therefore be disrupted in mice lacking the Dact1 protein. We tested this hypothesis using a *Dact1* mutant allele that we have described previously: This mutation was engineered via homologous recombination at the *Dact1* locus, generating a targeted allele (*Dact1^flox^*) in which the critical exon 2 is flanked by *loxP* sites. After excision by Cre recombinase this creates a frame-shift in the Dact1 transcript and results in complete loss of the Dact1 protein [25]. Descendants of mice in which this Cre-mediated event was transgenically driven in the germ-line therefore carry a constitutively *null* mutation in *Dact1* (*Dact1^−^*); animals homozygous for this allele are hereafter referred to as constitutive *Dact1* mutants.

**Figure 2:**
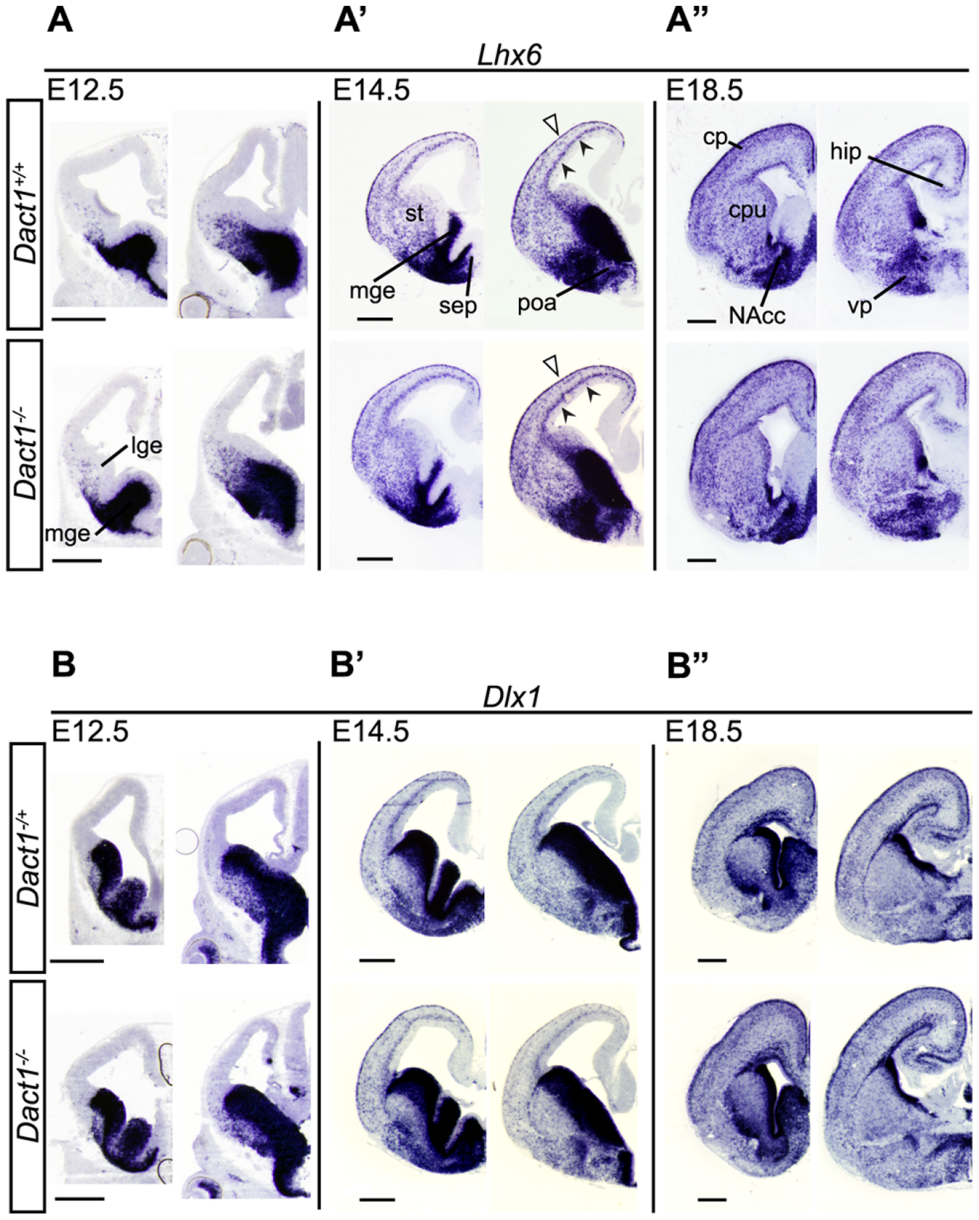
*Dact1^−/−^* mice have no major defects in the migratory paths of ganglionic eminence-derived GABAergic interneurons. *In situ* hybridization (Lhx6) in rostral to caudal coronal sections through E12.5 (**A**), E14.5 (**A**'), and E18.5 (**A**″) *Dact1^−/−^* brains (bottom) compared to wild type (top). Arrowheads indicate Lhx6 expression in the subventricular zone (closed) and marginal zone (open) of the cortex. *In situ* hybridization (Dlx1) in E12.5 (**B**), E14.5 (**B**'), and E18.5 (**B**″) *Dact1^−/−^* mice (bottom) compared to wild type (top). cpu, caudate-putamen; cp, cortical plate; hip, hippocampus; lge, lateral ganglionic eminence; mge, medial ganglionic eminence; NAcc, nucleus accumbens; poa, preoptic area; se, septum; st, striatum; vp, ventral pallidum. Scale bars = 500 µm.

To test for effects of Dact1 loss on the numbers and migration of these cells, we first examined the mRNA expression of Lhx6 and Dlx1, genes that mark tangentially migrating interneurons [32], in the developing forebrains of constitutive Dact1 mutant animals. We performed ISH on coronal brain sections from E12.5, E14.5, and E18.5 mice, comparing constitutive *Dact1* mutants to controls (Figure 2). Lhx6, a LIM-homeodomain transcription factor, is expressed in most MGE-derived neurons; it promotes the tangential migration of interneurons and regulates their differentiation and laminar distribution [43], [44], [45], [46]. We observed no change in the expression pattern of Lhx6 mRNA in the telencephalon of E12.5, E14.5, and E18.5 constitutive *Dact1* mutant mice (Figure 2A, A', A″). Similarly, the expression pattern of Dlx1 mRNA, which marks MGE and CGE-derived interneurons and their precursors, showed no changes (Figure 2B, B', B″); nor did the immunolocalization pattern of Calbindin protein, a general GABAergic neuron marker (data not shown). These data strongly suggest that there are no major defects in the production, tangential migration, or late gestational laminar positioning of cortical interneurons in constitutive *Dact1* mutants.

### Cultured cortical interneurons from constitutive *Dact1* mutant mice have fewer excitatory synapses

**Figure 3:**
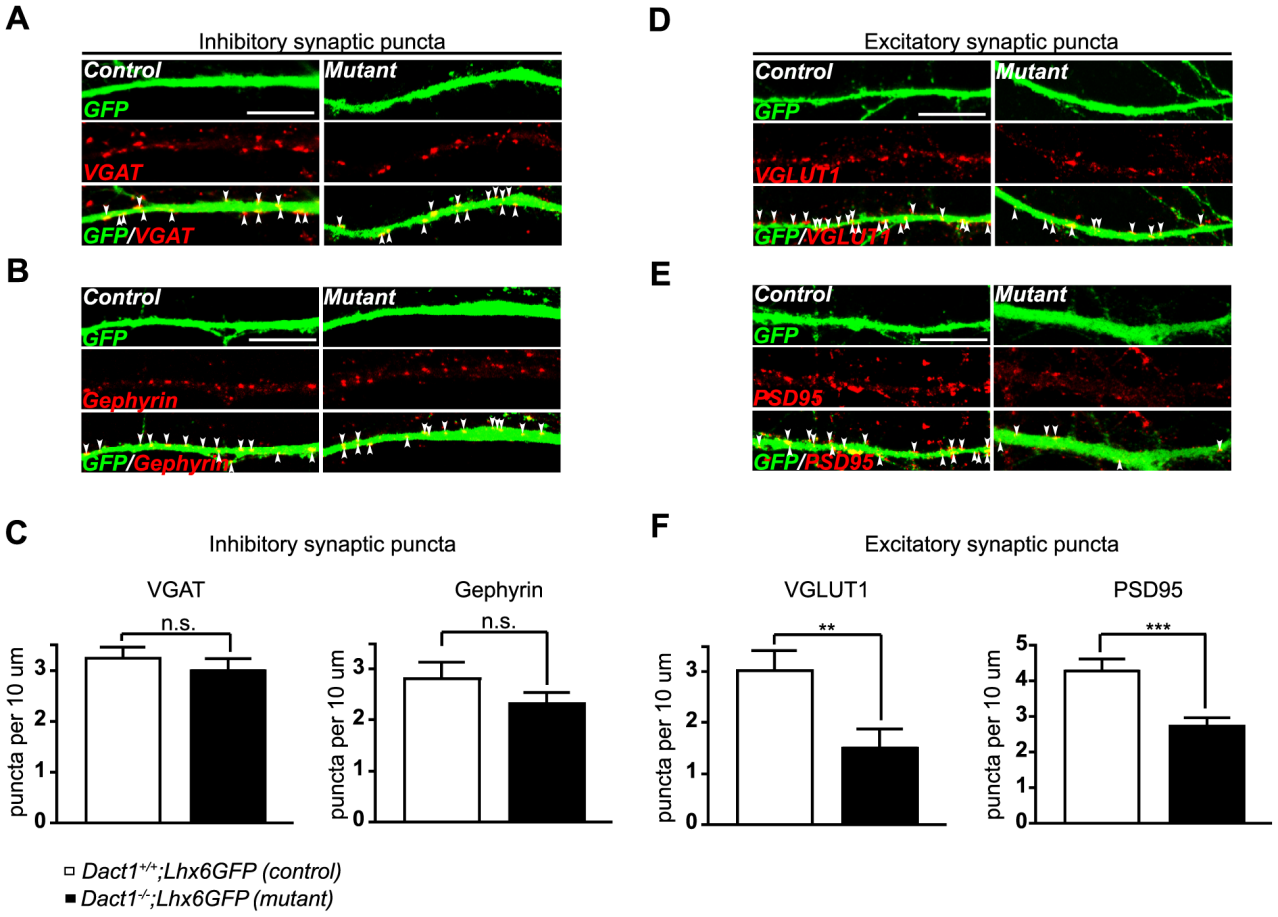
Cortical interneurons from constitutive *Dact1* mutant mice have fewer excitatory synapses on primary dendrites. Primary cortical cultures were prepared from postnatal day 0 *Dact1^−/−^;Lhx6GFP* (right) and control (left) brains, fixed at day *in vitro* 15, and synaptic puncta counted along GFP labeled primary dendrites from the cell soma to the first major branch point (arrowheads). Inhibitory synaptic puncta were visualized using antibodies against VGAT (presynaptic, **A**) and Gephyrin (postsynaptic, **B**) with each marker counted irrespective of co-localization with the other; **C** Quantification per 10 µm of primary dendrite length in control (open bars) and constitutive *Dact1* mutant neurons (closed bars). Excitatory synaptic puncta were visualized using antibodies against VGLUT1 (presynaptic, **D**) and PSD95 (postsynaptic, **E**) with each marker counted irrespective of co-localization with the other; **F** Quantification per 10 µm of primary dendrite length. Data shown are mean ± sem of at least 3 independent experiments, collected from at least 3 mice per genotype, 10–15 neurons per animal. **p<0.01; ***p<0.001; n.s., not significant. Scale bars = 10 µm.

Given the expression of *Dact1* in immature cortical interneurons and its continued expression in the cortex of adult mice [26], we sought to determine if Dact1 is important for the maturation of interneurons as they integrate into the cortical circuitry. This goal was also driven by our previous studies, which revealed Dact1 enrichment in postsynaptic fractions and its requirement in pyramidal neurons for spine and excitatory synapse formation [11]. To test whether Dact1 might play a similar role in maturing interneurons, we crossed the *Dact1* mutant line to a BAC transgenic *Lhx6GFP* line [32], thereby facilitating identification of GE-derived interneurons. High density primary neuronal cultures were prepared from postnatal day 0 (P0) cortices of *Dact1^−/−^;Lhx6GFP* and *Dact1^+/+^;Lhx6GFP* littermate mice and cultured for 15 days *in vitro* (DIV). When we visualized inhibitory synapses by staining with antibodies against vesicular GABA transporter (VGAT), a presynaptic inhibitory marker (Figure 3A), and Gephyrin, a postsynaptic inhibitory marker (Figure 3B), there was a non-significant trend towards reduction in *Lhx6GFP* ^+^ cultured neurons from constitutive *Dact1* mutants (VGAT puncta: 3.00±0.22 per 10 µm in *Dact1^−/−^* versus 3.24±0.22 in control, *p* = 0.4480; Gephyrin puncta: 2.32±0.22 per 10 µm in *Dact1^−/−^*, versus 2.81±0.32 in control, *p* = 0.2934) (Figure 3C). In contrast, when we visualized excitatory synapses by staining with antibodies against vesicular glutamate transporter 1 (VGLUT1), a presynaptic excitatory marker (Figure 3D), and postsynaptic density-95 (PSD95), a postsynaptic excitatory marker (Figure 3E), there was a significant reduction in both the pre- (by∼50%) and post-synaptic (by ∼36%) excitatory markers along primary proximal dendrites of *Lhx6GFP^+^* mutant interneurons (VGLUT1 puncta: 1.50±0.36 per 10 µm in *Dact1^−/−^* versus 3.02±0.38 in control, *p* = 0.0084; PSD95 puncta: 2.74±0.22 per 10 µm in *Dact1^−/−^* versus 4.27±0.33 in control, *p* = 0.0005) (Figure 3F).

In summary, our data in primary neuronal cultures derived from mice constitutively *null* for Dact1 support a role for this protein in the formation of synapses, specifically excitatory synapses, on cortical interneuron dendrites. However, this finding is confounded by the fact that in these cultures all cells lack *Dact1*. This leaves open the possibility that interneuron phenotypes in these cultures might be non-cell-autonomous. That is, they may occur secondary to a requirement for Dact1 in co-cultured excitatory neurons, especially because we have previously shown that *Dact1* mutant excitatory neurons have dendrite, spine, and synapse phenotypes [11]. We next used conditional mutagenesis to circumvent this potential confound.

### Dact1 is required cell-autonomously and postsynaptically in interneurons for the formation of excitatory inputs

**Figure 4:**
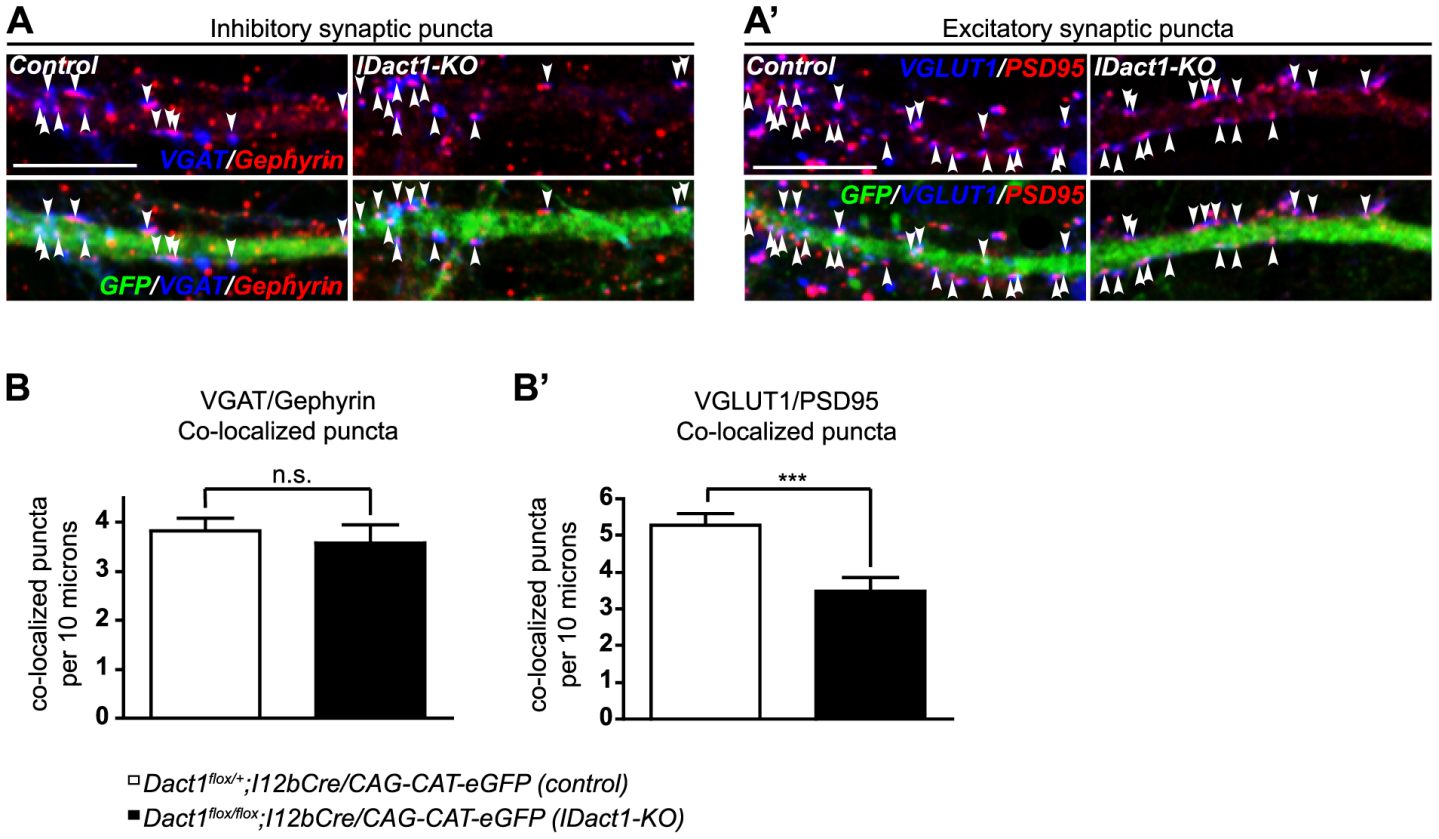
Reduction of excitatory synapses in *Dact1* mutant cortical interneurons is cell-autonomous. Primary cortical cultures were prepared from postnatal day 0 Interneuron-specific *Dact1* mutant (*IDact1-KO*) (right) and control (left) brains, fixed at day *in vitro* 15, and pre- and post-synaptic co-localized puncta counted along GFP labeled primary dendrites from the cell soma to the first major branch point (arrowheads). VGAT/Gephyrin (inhibitory, **A**) and Vglut1/PSD95 (excitatory, **A**') co-localized puncta (arrowheads) along the GFP+ dendrite in control (left) and *IDact1-KO* (right) mice. Quantification of co-localized inhibitory (**B**) and excitatory (**B**') pre- and postsynaptic puncta in control (open bars) and *IDact1-KO* mutants (closed bars). Data shown are mean ± sem of at least 2 independent experiments, collected from at least 2 mice per genotype, 10–15 neurons per animal. ***p<0.001; n.s., not significant. Scale bars = 10 µm.

To determine if the synapse phenotypes on interneuron dendrites in constitutive *Dact1* mutant cultures were due to a cell-autonomous requirement for Dact1, we made use of the *Dact1^flox^* allele [25] in combination with a *Dlx*-enhancer element-Cre (*I12b-Cre*) transgenic allele that drives Cre expression specifically in interneurons starting at pre-migratory stages [29]. Mice homozygous for the targeted *Dact1^flox^* allele and carrying *I12b-Cre* excise *Dact1* exon2 and thereby lose the Dact1 protein specifically in interneurons. To visualize Cre^+^ interneurons within Cre^−^ wild type tissues derived from these mice, we crossed into this line a transgenic Cre-dependent eGFP reporter, *CAG-cat-eGFP* [30], in which eGFP is expressed upon Cre-mediated excision of the loxP-flanked CAT gene. From this point forward we refer to these mice as *IDact1-KO* (Interneuron-specific Dact1 Knockout) mutants. High density neuronal cultures were prepared from cortices of P0 *IDact1-KO* (*Dact1^flox/flox^;I12bCre/CAG-cat-eGFP*) and littermate control (*Dact1^flox/+^;I12bCre/CAG-cat-eGFP*) mice and cultured *in vitro* for 15 days. Co-localized pre- and post-synaptic puncta were then counted along the primary dendrites of GFP labeled interneurons. As in the constitutive *Dact1* mutant cultures, inhibitory synapses along GFP-labeled interneuron dendrites in *IDact1-KO* cultures were not significantly reduced in number, showing only a modest trend in this direction (co-localized inhibitory pre-synaptic (VGAT) and post-synaptic (Gephyrin) puncta: 3.58±0.36 per 10 µm in *IDact1-KO* versus 3.82±0.25 in control, *p* = 0.5847) (Figure 4A, B). Also consistent with findings in constitutive *Dact1* mutant cultures, excitatory synapses were significantly reduced (by ∼34%) on GFP-labeled *IDact1-KO* interneuron dendrites compared to controls (co-localized excitatory pre-synaptic (VGLUT1) and post-synaptic (PSD95) puncta: 3.48±0.38 per 10 µm in *IDact1-KO* versus 5.28±0.31 in control, *p* = 0.0006) (Figure 4A', B'). We replicated this finding in additional *IDact1-KO* cultures in which pre- and post-synaptic puncta were quantified separately, irrespective of co-localization (method as in Fig 3) (Figure S1).

In summary, we found significant reductions in the number of excitatory synapses on cultured cortical interneurons from *IDact1-KO* mutants, in which only interneurons have lost Dact1, while the number of inhibitory synapses on these dendrites showed a non-significant trend toward reduced numbers. These data support a cell-autonomous role for Dact1 in the formation of excitatory synapses in developing cortical interneurons. This is particularly significant in light of our prior results showing that Dact1 mutant cortical pyramidal neurons have concomitant reductions in excitatory synapses and in dendritic spines [11]. Since the interneurons we characterized in the present study do not have spines, the synapse reductions we observe on their dendrites cannot reflect a secondary consequence of spine loss; these data instead provide strong support for a distinct role for Dact1 in synapse formation.

### Inhibitory neurons in *IDact1-KO* mice have reduced synapse numbers along primary dendrites *in vivo*

**Figure 5:**
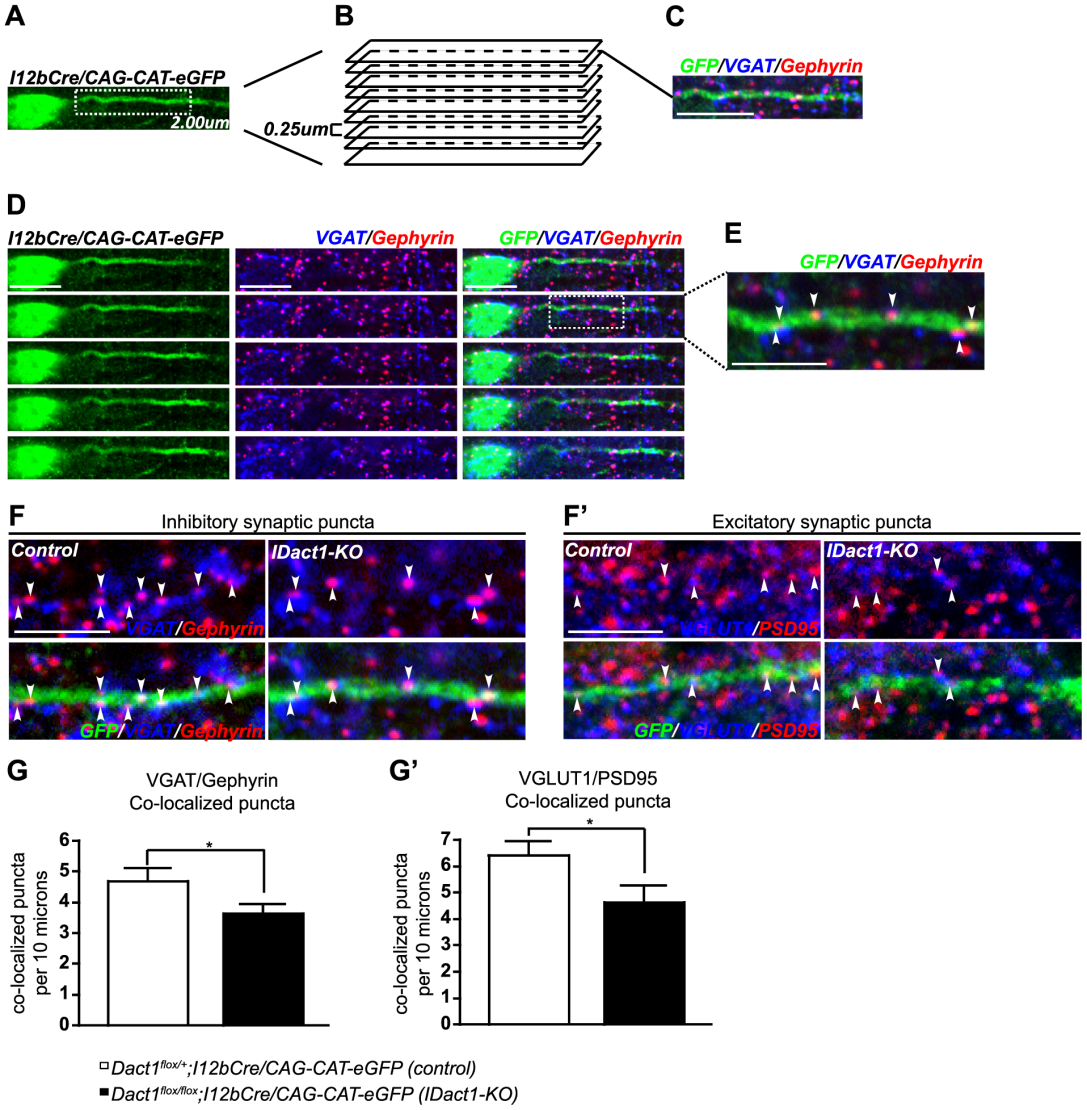
Interneuron-specific *Dact1* mutant mice (*IDact1-KO*) have fewer excitatory and inhibitory synapses along cortical interneuron dendrites. **A-E** Experimental design. **A** Composite z-stack image of a GFP+ interneuron located in upper layers of the primary somatosensory cortex, selected for synaptic puncta quantification from postnatal day 30 mouse brain sections. **B** This representative composite z-stack image is composed of 9 optical sections, 0.25 microns apart, encompassing the proximal dendrite. **C** Example of optical section used to confirm pre- and post-synaptic co-localization along the GFP+ dendrite. **D** Representative serial z-stack images used to confirm synaptic puncta co-localization in each z-plane. Co-localized puncta visible in adjacent serial sections are scored only once to avoid duplicate counting of individual synapses. **E** Boxed region is shown at the higher magnification, as employed to identify: VGAT/Gephyrin (inhibitory, **F**) and Vglut1/PSD95 (excitatory, **F**') co-localized puncta (arrowheads) along the GFP+ dendrite in control (left) and *IDact1-KO* (right) mice. Quantification of co-localized inhibitory (**G**) and excitatory (**G**') pre- and post-synaptic puncta in control (open bars) and *IDact1-KO* mutants (closed bars). Data shown are mean ± sem of at least 3 independent experiments, collected from at least 3 mice per genotype, 5-10 neurons per animal. *p<0.05. Scale bars = 5 µm.

Since dissociated cultures do not fully recapitulate normal development, we next studied interneuron dendrite and synapse development in the brains of *IDact1-KO* mice. To accomplish this, we made 20 micron coronal sections from P30 *IDact1-KO* and control mice and then identified Cre-mediated GFP labeled interneurons from upper layers of the primary somatosensory cortex. We collected composite confocal images, comprised of a series of z-stack slices encompassing proximal dendrites of GFP labeled interneurons (Figure 5A–C). Representative serial z-stacks were then used to confirm pre- and post-synaptic puncta co-localization along a single GFP labeled dendrite in each z-plane (Figure 5D,E). Interestingly, compared to results obtained in primary culture, *in vivo* there was a more pronounced and significant decrease (by ∼23%) in the number of inhibitory synapses along dendrites of mutant interneurons compared to controls (co-localized inhibitory pre-synaptic (VGAT) and post-synaptic (Gephyrin) puncta: 3.62±0.30 per 10 µm in *IDact1-KO* versus 4.69±0.43 in control, *p* = 0.0471) (Figure 5F, G). For excitatory synapses, the results obtained *in vivo* were similar to *in vitro*: there were significant reductions (by ∼28%) in the number of excitatory synapses along dendrites of mutant interneurons compared to controls (co-localized excitatory pre-synaptic (VGLUT1) and post-synaptic (PSD95) puncta: 4.62±0.64 per 10 µm in *IDact1-KO* versus 6.42±0.52 in control, *p* = 0.0368) (Figure 5F', G'). These results demonstrate that there are fewer inhibitory as well as excitatory synapses on the dendrites of interneurons within the cortices of *IDact1-KO* mice.

### Reduced excitatory synapse numbers are rescued in *IDact1-KO* interneurons by specific lentivirus-mediated re-expression of Dact1, Dvl1, or DISC1

**Figure 6:**
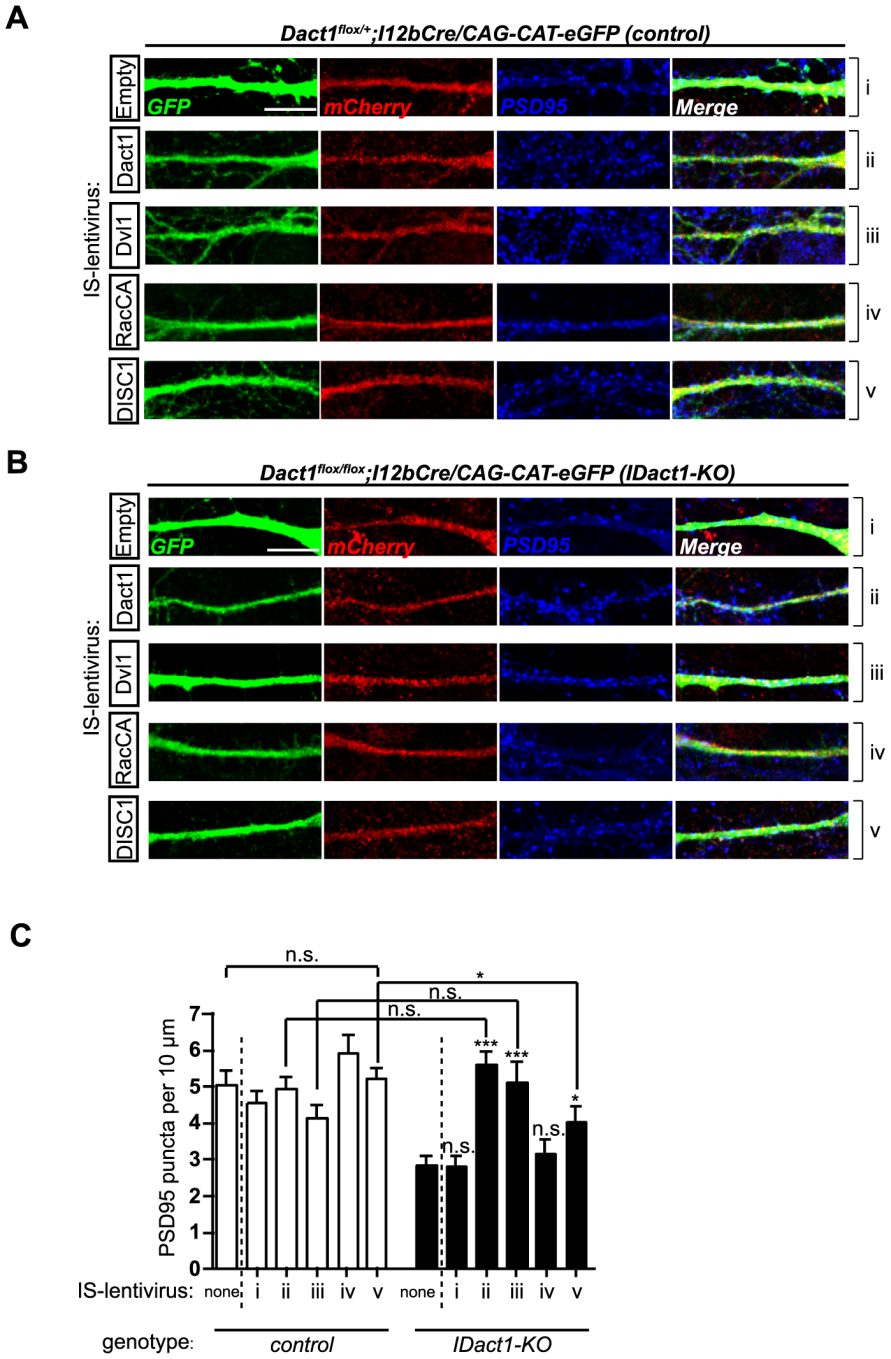
Interneuron-specific expression of Dact1, Dvl1, or DISC1 rescues excitatory synapse numbers in *IDact1-KO* interneurons. **A–B** Infected interneurons were detected by expression of mCherry. Characterization of PSD95 puncta on primary dendrites of cultured GFP+ cortical interneurons from control (**A**) and *I-Dact1KO* (**B**) mice infected with IS-lentiviral constructs expressing (**i**) mCherry alone, (**ii**) Dact1, (**iii**) Dvl1, (**iv**) RacCA, or (**v**) DISC1. C Quantification of PSD95 puncta per 10 µm of primary dendrite length in control (open bars) and *IDact1-KO* mutant neurons (closed bars). Results are presented as mean ± sem from at least 2 independent experiments, collected from at least 2 mice per genotype, 10–15 neurons per animal. *p<0.05; ***p<0.001; n.s., not significant. All *p* values are relative to either control (IS-lentivirus, none) or *IDact1-KO* (IS-lentivirus, none). Scale bars = 10 µm.

We next sought to determine if recombinant expression of Dact1 was sufficient to restore synapse numbers in *IDact1-KO* interneurons. To achieve this, we constructed a lentiviral vector containing a Dlx1/2-I12b interneuron specific enhancer [47] driving mCherry expression 5′ to a T2A peptide and multiple cloning site (MCS), henceforth referred to as Interneuron-Specific (IS)-lentivirus. To confirm that this vector drives specific expression in GABAergic neurons, neuronal cultures were prepared from P0 cortices of *GAD1-GFP* mice in which GABA^+^ interneurons can be visualized by GFP expression (Figure S2). Cells were infected at DIV 1 with a lentiviral construct containing either a CMV promoter or Dlx1/2-I12b interneuron specific enhancer driving mCherry expression. Cells were then fixed and stained 5 days later. In *GAD1-GFP* cultures infected with a control lentiviral construct containing the CMV promoter driving mCherry, a wide variety of cells expressed mCherry including interneurons co-labeled with GFP but also many non-GFP^+^ cells such as pyramidal neurons and astrocytes (Figure S2A, top row). In contrast, in *GAD1-GFP* cultures infected with the IS-lentivirus, all cells expressing mCherry co-labeled with *GFP*, confirming that the IS-lentivirus drives recombinant gene expression specifically in GABAergic interneurons (Figure S2A, bottom row). We also confirmed expression of each recombinant cDNA subcloned into this lentiviral vector by Western Blot analyses of the corresponding FLAG or HA-tagged proteins in infected HEK293T cells (Figure S2B) and by immunocytochemistry in infected cultured neurons (Figure S2C). As a final validation of control conditions in our experimental design, high density neuronal cultures were prepared from P0 *IDact1-KO* and control cortices, infected with IS-lentiviruses at DIV10, fixed at DIV15, and excitatory synapse numbers quantified using PSD95 as a marker. There were no significant differences between uninfected wild type control neurons and wild type control neurons infected with any of the recombinant IS-lentivirus constructs, demonstrating that each of the proteins we tested for rescue does not produce dominant effects on synapse numbers when recombinantly expressed under these conditions (Figure 6A, C open bars). Excitatory synapse numbers were significantly reduced (by ∼38%) in empty IS-lentivirus infected *IDact1-KO* neurons compared to wild type neurons (Figure 6A, Bi, C closed bar i *vs*. open bars). This was extremely consistent with our prior results in uninfected *IDact1KO* interneurons, and confirmed that infection with empty IS-lentivirus in itself had no effect on synapse numbers.

Using this novel molecular strategy, we found that interneuron-specific expression of mouse Dact1 restored PSD95 puncta numbers to wild type levels in *IDact1-KO* interneurons (PSD95 puncta: 5.59±0.35 per 10 µm in *IDact1-KO* + IS-lentivirus (Dact1), versus 2.85±0.24 in *IDact1-KO* + IS-lentivirus (empty), *p*<0.0001) (Figure 6B, C closed bar ii *vs*. i). This demonstrated that *in vitro*, recombinant re-expression of Dact1 within *Dact1* mutant interneurons is sufficient to rescue excitatory synapse numbers on dendrites, corroborating that the requirement for Dact1 in synapse formation is cell-autonomous at the postsynapse.

Dact proteins have been reported to functionally interact with a diverse array of partners ranging from cytoplasmic Wnt pathway components to transmembrane proteins and even nuclear factors [21], [22], [24], [25], [34]. We accordingly sought to determine if any of these previously proposed partners function with Dact1 at the postsynapse. The cytoplasmic Dvl1 protein is a direct binding partner of Dact1 [21], [34], is a core Wnt pathway component [48], and has been shown to regulate both presynaptic assembly and the postsynaptic formation of excitatory synapses [17], [20]. Similar to rescue with Dact1 itself, recombinant expression of Dvl1 in *IDact1-KO* interneurons fully rescued excitatory synapse numbers (PSD95 puncta: 5.11±0.57 per 10 µm in *IDact1-KO* + IS-lentivirus (Dvl1), versus 2.85±0.24 in *IDact1-KO* + IS-lentivirus (empty), *p*<0.0001) (Figure 6B, C closed bar iii *vs*. i).

In previous work, we showed that Dact1-deficient pyramidal neurons have reductions in activity of the small GTPase Rac, and that recombinant expression of a constitutively active form of this protein (RacCA) rescues spine phenotypes in these cells [11]. Interestingly and in stark contrast to the rescue with either Dact1 or Dvl1, recombinant expression of RacCA had no effect on synapse numbers in Dact1-deficient interneurons (PSD95 puncta: 3.16±0.38 per 10 µm in *IDact1-KO* + IS-lentivirus (RacCA), versus 2.85±0.24 in *IDact1-KO* + IS-lentivirus (empty), *p* = 0.4738) (Figure 6B, C closed bar iv *vs*. i). This provides further support for the conclusion, already suggested by our phenotypic evidence in aspiny interneurons, that the role for Dact1 in synapse formation is molecularly distinct from its role in dendrite and spine morphogenesis.

Finally, we sought to implicate additional proteins in a Dact1-Dvl1 pathway at the postsynapse. To this end we tested whether recombinant expression of the Disrupted in Schizophrenia-1 (DISC1) scaffold protein could rescue *Dact1* mutant synapse phenotypes. DISC1 localizes to the postsynaptic compartment of excitatory synapses [49], [50] and has been shown to cooperate with another Dvl-binding protein, Dixdc1, in the regulation of Wnt and other neurodevelopmental signaling pathways [51], [52]. Provocatively, recombinant expression of mouse DISC1 in *IDact1-KO* interneurons partially rescued excitatory synapse numbers (PSD95 puncta: 4.04±0.42 per 10 µm in *IDact1-KO* + IS-lentivirus (DISC1), versus 2.85±0.24 in *IDact1-KO* + IS-lentivirus (empty), *p* = 0.0143) (Figure 6B, C closed bar v *vs*. i).

To summarize, rescue of synapse numbers in *IDact1-KO* interneurons was complete for lentiviral-mediated expression of either Dact1 or Dvl1 (PSD95 puncta: 5.59±0.35 per 10 µm in *IDact1-KO* + IS-lentivirus (Dact1), versus 4.89±0.27 in control + IS-lentivirus (Dact1), p = 0.1209; PSD95 puncta: 5.11±0.57 per 10 µm in *IDact1-KO* + IS-lentivirus (Dvl1), versus 4.15±0.34 in control + IS-lentivirus (Dvl1), *p* = 0.2093) (Figure 6A, B, C closed *vs*. open bars for ii & iii respectively). In contrast, rescue by DISC1, while significant compared to control mutant interneurons, was not complete: synapse numbers in DISC1-rescued *Dact1* mutant interneurons remained significantly reduced (by ∼23%) when compared to wild type controls (PSD95 puncta: 4.04±0.42 per 10 µm in *IDact1-KO* + IS-lentivirus (DISC1), versus 5.22±0.29 in control + IS-lentivirus (DISC1), *p* = 0.0291) (Figure 6A, B, C closed *vs*. open bar for v). Finally, constitutively active Rac, which robustly rescues spine phenotypes in Dact1-deficient pyramidal neurons [11], is insufficient to rescue synapse phenotypes in Dact1-deficient interneurons as already described above (Figure 6B, C closed bar iv *vs*. i or ‘none’).

## Discussion

### Cell autonomous role for Dact1 within the postsynaptic compartment of cortical interneurons

Dact1 is expressed in Dlx-dependent cortical interneurons that tangentially migrate from their place of birth in the ganglionic eminences to the neocortex. Nonetheless, loss of Dact1 alone does not significantly affect the migration of these GE-derived interneurons as there are no major defects in tangential migration, distribution, or laminar position of these cells in the neocortex of mice homozygous for a *null* mutation in *Dact1*.

The most abundant neuron type in the mammalian cortex is the pyramidal projection neuron [53], [54], and excitatory postsynaptic connections on pyramidal neuron dendrites are located primarily on morphologically plastic dendritic spines. It is therefore not surprising that mammalian cortical synaptogenesis has been largely studied at excitatory synapses in conjunction with spine formation in pyramidal neurons [55]. In contrast, synaptogenesis in the less abundant cortical interneurons, which have few or no spines, is just beginning to be elucidated [56], [57]. The data presented here support a role for Dact1 within the postsynaptic compartment of cortical interneurons. Our loss-of-function genetic analyses demonstrate that Dact1 is required in maturing cortical interneurons for synapse development. Cortical interneurons cultured from constitutive *Dact1* mutants have fewer numbers of excitatory synaptic puncta along primary dendrites. Importantly, synapse phenotypes in *Dact1* mutant interneurons are cell-autonomous and do not occur secondary to loss of Dact1 in excitatory presynaptic partners. This is proven by our data using Interneuron-Specific *Dact1* conditional mutants, which shows that cortical interneurons have fewer excitatory synapses on their dendrites, *in vitro* or *in vivo*, despite the presence of genotypically wild type excitatory partners. This conclusion is rendered incontrovertible by our further experiment demonstrating that this synapse phenotype is fully rescued by re-expression of Dact1 specifically within interneurons using a novel interneuron-specific lentiviral vector.

### Differences between synapse phenotypes *in vitro* versus *in vivo*

In contrast to our results in DIV15 dissociated cultures, interneuron-specific loss of Dact1 resulted in significant reductions in both excitatory and inhibitory synapses along cortical interneuron dendrites in P30 brain tissue. We noted a trend toward reduced inhibitory synaptic markers in our *in vitro* studies both here and previously [11], supporting a function for Dact1 at both the inhibitory and excitatory postsynapse. Nonetheless, the more robust and significant loss of inhibitory synapses in brain tissue compared to dissociated neuronal cultures suggests, among other possibilities, that observed effects on inhibitory synapse numbers might take longer to develop and be indirect, perhaps mediated through circuit mechanisms. To elaborate, a single neuron of any class receives both excitatory and inhibitory inputs from diverse synaptic partners that are plastic; in the functioning brain the strength and numbers of these synapses must therefore be continuously modulated to maintain a proper balance of excitation and inhibition [58], [59]. On this basis, a cell-autonomous effect on the formation of excitatory inputs could lead to secondary changes in inhibitory inputs downstream of both cellular and network mechanisms that homeostatically maintain excitatory/inhibitory input balance on dendrites. A reasonable hypothesis that would be consistent with our experimental findings is that such homeostatic mechanisms take longer to occur and may also require cell-cell and circuit interactions that are not fully replicated in DIV15 dissociated cultures, resulting in less robust effects on inhibitory synapse numbers in this *in vitro* system compared to effects in more mature brain tissue with fully intact neuronal and glial relationships.

### Low levels and potential functional redundancy of Dact paralogs

There are two major molecular caveats to the conclusions discussed above. The first is that we were unable to biochemically demonstrate (*i.e*. at the protein level) Cre-mediated loss of Dact1 specific to *IDact1-KO* interneurons. We attribute this to a technical limitation: Although several commercial antibodies and our own custom-made antibodies readily detect the Dact1 protein when it is recombinantly expressed, repeated experience in the Cheyette laboratory has been that these reagents are unable to accurately detect the endogenous Dact1 protein in many tissues where the mRNA is expressed. Despite this difficulty, in a tissue where the Dact1 mRNA is most abundant (adult uterus), we have previously demonstrated that the Cre-excised *Dact1* mutant allele (*Dact1^−^*) derived from *Dact1^flox^* (as employed here to produce conditional mutant interneurons) is *null* for Dact1 protein [25]. Although the loss of Dact1 protein in *IDact1-KO* interneurons is inferred, the close agreement of our phenotypic findings in labeled interneurons from *Dact1* ^−/−^ (constitutive) and *IDact1-KO* (conditional) mutants, as well as the rescue of these phenotypes via lentiviral mediated interneuron-specific re-expression of Dact1, strongly support that *IDact1-KO* interneurons are deficient for Dact1 protein and that this is the molecular cause for the synapse phenotypes we observe.

The most parsimonious explanation for our published and unpublished findings regarding Dact1 protein expression is that in most tissues where Dact1 is functional, including in developing neurons, physiological levels are too low to detect via conventional antibody-based methods such as immunoblotting or immunohistology. Such very low levels in normal cells may reflect pathophysiological consequences when the protein is present at higher concentrations [34]. In the case of the postsynapse, very low protein levels may also reflect the extremely limited molecular space in this subneuronal compartment, optimized to achieve maximum signaling efficiency and plasticity with minimum membrane area and cytoplasmic volume.

One Dact family member has been detected independently via mass spectrometry as an endogenous component of the mammalian postsynaptic density: Dact3 [60]. This introduces the second molecular caveat to our present work: Potential functional redundancy between the three mammalian Dact family members. It is possible, for example, that the lack of observed defects in interneuron production or migration in *Dact1* mutant animals reflects functional substitution by the Dact2 and Dact3 proteins that have overlapping neurodevelopmental expression patterns [26] and that remain unaltered in these animals. In preliminary experiments employing animals simultaneously mutant for two Dact paralogs (*i.e*. *Dact1^−/−^; Dact3^−/−^ KO* mice) we have observed no effects on interneuron numbers or migration (data not shown). Future work in the Cheyette laboratory will examine the question of neurodevelopmental redundancy in animals in which all three Dact family members have been conditionally eliminated in this and other neuronal populations.

### Function of Dact1 at the postsynapse

The postsynapse is a highly organized subcellular compartment containing complexes of neurotransmitter receptors and other transmembrane proteins, scaffold proteins, and signaling components [10], [61], [62]. Given that Dact1 is enriched in this compartment [11], how does it function there? Dact1 has a C-terminal PDZ-binding motif [21], [26], and PDZ domain proteins and their binding partners are important for the organization and assembly of molecular complexes [61]. Such molecular complexes at the postsynaptic density (PSD) of excitatory synapses contribute not only to synapse structure but also to strength and plasticity of synaptic activity [63]. As a PDZ-binding protein, Dact1 may function in the recruitment and stabilization of intracellular PDZ partners at the PSD. Synaptically localized cell adhesion molecules that interact with scaffold proteins also have well-established roles at the synapse [61]. In the vertebrate embryo Dact1 has been reported to bind and stabilize the cytoplasmic p120-catenin protein [24] that itself interacts with and stabilizes transmembrane cadherin proteins [64], [65]. As we have previously shown for Dact1 [11], in glutamatergic neurons p120-catenin operates upstream of Rho GTPases such as Rac that regulate cytoskeletal dynamics contributing to dendrite and spine development [66]. Activation of Rac1 in glutamatergic neurons can initiate the formation of dendritic spines and also induce the postsynaptic clustering of AMPA receptors [67]. P120-catenin has also been proposed to influence synapse formation by promoting cadherin stability at the synapse [55], [62]. On this basis, Dact1 may affect synapse numbers through the postsynaptic regulation of p120-catenin, associated cadherins, and Rac activity. Arguing against this hypothesis, recombinant expression of either RacCA (Fig 6) or p120-catenin (data not shown) fails to rescue synapse numbers in *IDact1-KO* interneurons.

Dact1 has previously been proposed to modulate Wnt signaling via direct interactions with Dvl proteins [21], [22]. Dvl proteins are Wnt signaling hubs [48] that help regulate dendrite morphology [68] and the assembly of presynaptic terminals [13], [69]. Dvl1 is also present at the postsynapse where it is required for Wnt7a signaling to promote the formation of excitatory synapses [20]. Consistent with a role for Dact1 at the postsynapse upstream or in conjunction with Dvl proteins, recombinant expression of Dvl1 rescues excitatory synapse numbers on the dendrites of *IDact1-KO* interneurons. Interestingly, these results argue against some previous models proposing that Dact1 physiologically promotes Dvl degradation [23]; our rescue data suggests instead that at the postsynapse Dact1 functions in the recruitment or stabilization of Dvl to support synapse formation.

Like Dvl1, which serves as a hub for the Wnt signaling pathways throughout development, DISC1 serves as a hub for several signaling pathways implicated in neurodevelopmental processes [70], [71]. DISC1 interacts with Dixdc1, a Dvl-binding protein, to mediate Wnt-dependent and Wnt-independent signaling pathways in developing neurons [52]. Among other subcellular locations, DISC1 is present in the PSD of excitatory synapses where it is important for regulating synapse formation and function [49], [50]. We show here that recombinant expression of DISC1 partially rescues synapse phenotypes in *IDact1-KO* interneurons. Provocatively, when recombinantly expressed in HEK293T cells, Dact1 can form a complex with DISC1 (Figure S3A). Taken together with our prior results in pyramidal neurons, our data support a model (Figure S3B) in which Dact1 has at least two distinct roles in maturing neurons: 1) a Rac-dependent role, possibly through p120-catenin, in promoting actin and other cytoskeletal rearrangements necessary for dendrite and spine formation [11], and 2) a Dvl-dependent role, either in conjunction with or in parallel to DISC1, in the postsynaptic compartment for synapse formation. Either of these roles could occur downstream of intercellular communication mediated by Wnt ligand activated transmembrane receptor complexes. Future experimental work in the Cheyette laboratory will explore functional hypotheses generated by this molecular model.

Given the findings reported here, in the future it will be valuable to examine how the electrophysiological activity of synapses and circuits are altered in the brains of *IDact1-KO* mutant animals, as well as in other conditional mutant mouse lines with selective ablation of Dact1 in different neuron classes. Downstream behavioral consequences in these animals could be of considerable translational interest, as excitatory:inhibitory synapse balance has been proposed as a pathogenic mechanism in major mental disorders, including those associated with disruption of *DISC1* [2], [5], [58].

## Supporting Information

Figure S1 **Reduction of excitatory synapses in** ***Dact1*** **mutant cortical interneurons is cell-autonomous (independent replication).** Primary cortical cultures were prepared from postnatal day 0 Interneuron-specific *Dact1* mutant (*IDact1-KO*) (right) and control (left) brains, then processed and analyzed as in Figure 3 with pre- and post-synaptic markers counted irrespective of colocalization with each other. Inhibitory synaptic markers: VGAT (presynaptic, **A**), Gephyrin (postsynaptic, **B**). **C** Quantification in control (open bars) and *IDact1-KO* (closed bars) neurons. Excitatory synaptic markers: VGLUT1 (presynaptic, **D**), PSD95 (postsynaptic, **E**). **F**. Quantification. Data shown are mean ± sem of at least 3 independent experiments, collected from at least 3 mice per genotype, 10–15 neurons per animal. ***p<0.001; n.s., not significant. Scale bars = 10 µm.
(TIF)

Figure S2 **Interneuron specific (IS)-lentivirus drives specific expression in GABAergic interneurons.** **A** Neuronal cultures prepared from postnatal day 0 cortices from *GAD1-GFP* mice were infected with either a lentiviral construct containing a CMV promoter (top panel) or a Dlx1/2-I12b interneuron specific enhancer (bottom panel) driving mCherry expression. mCherry driven by the CMV promoter containing lentivirus labels some *GFP* ^+^ interneurons (yellow arrowheads, top panel) plus many non-*GFP* ^+^ cells (magenta arrowheads, top panel). mCherry driven by the interneuron specific enhancer containing lentivirus labels only *GFP* ^+^ interneurons (yellow arrowheads, bottom panel). **B** Human Embryonic Kidney 293T cells were transfected with IS-lentiviral constructs, collected at 3 days post-transfection, and lysates prepared and immunoblotted to confirm specific recombinant protein expression by the constructs used for synapse phenotype rescue experiments. **C** Neuronal cultures prepared from P0 cortices from wild type mice were infected with IS-lentiviral constructs at DIV1, fixed at DIV7, and stained with either FLAG or HA antibody to confirm recombinant protein expression levels. Scale bars = 100 µm.
(TIF)

Figure S3 **Model reflecting distinct roles for Dact1 in maturing neurons.** **A** Dact1 forms a complex with Disrupted in Schizophrenia-1 (DISC1) when co-expressed in an immortalized human cell line. FLAG-tagged murine Dact1 or HA-tagged murine DISC1 were recombinantly expressed in HEK293T cells, protein complexes immunoprecipitated (IP) with anti-FLAG agarose beads, and associated proteins detected by immunoblot (IB) with anti-HA antibody. **B** ***Left***: Dact1 promotes actin and other cytoskeletal rearrangements necessary for dendrite and spine formation through a Rac-dependent mechanism that may also involve p120-catenin. ***Right***: Within the postsynaptic compartment Dact1 acts with Dvl1 and possibly with DISC1 (dashed arrow) in synapse formation. ***Center***: Intercellular Wnt ligands and their transmembrane receptor complexes may operate upstream of one or both of these pathways.
(TIF)
