# Supplementary figures and images for: Dapper Antagonist of Catenin-1 Cooperates with Dishevelled-1 during Postsynaptic Development in Mouse Forebrain GABAergic Interneurons
Source: PLoS One. 2013 Jun 24;8(6):e67679. doi: 10.1371/journal.pone.0067679 (PMC3691262; doi:10.1371/journal.pone.0067679)

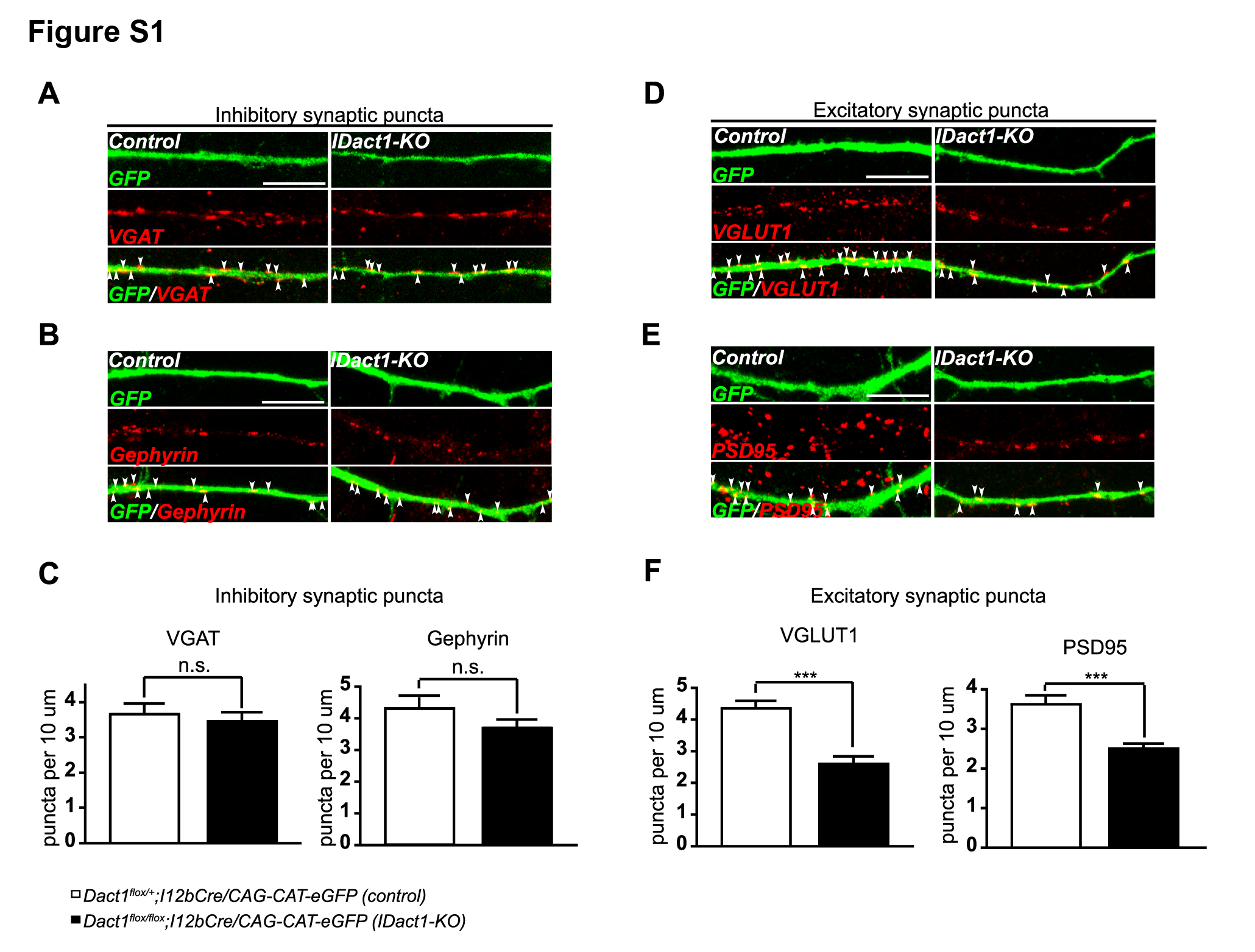

Supplement: Figure S1 — Reduction of excitatory synapses in Dact1 mutant cortical interneurons is cell-autonomous (independent replication). Primary cortical cultures were prepared from postnatal day 0 Interneuron-specific Dact1 mutant (IDact1-KO) (right) and control (left) brains, then processed and analyzed as in Figure 3 with pre- and post-synaptic markers counted irrespective of colocalization with each other. Inhibitory synaptic markers: VGAT (presynaptic, A), Gephyrin (postsynaptic, B). C Quantification in control (open bars) and IDact1-KO (closed bars) neurons. Excitatory synaptic markers: VGLUT1 (presynaptic, D), PSD95 (postsynaptic, E). F. Quantification. Data shown are mean ± sem of at least 3 independent experiments, collected from at least 3 mice per genotype, 10–15 neurons per animal. ***p<0.001; n.s., not significant. Scale bars = 10 µm. (TIF) [file pone.0067679.s001.tif]

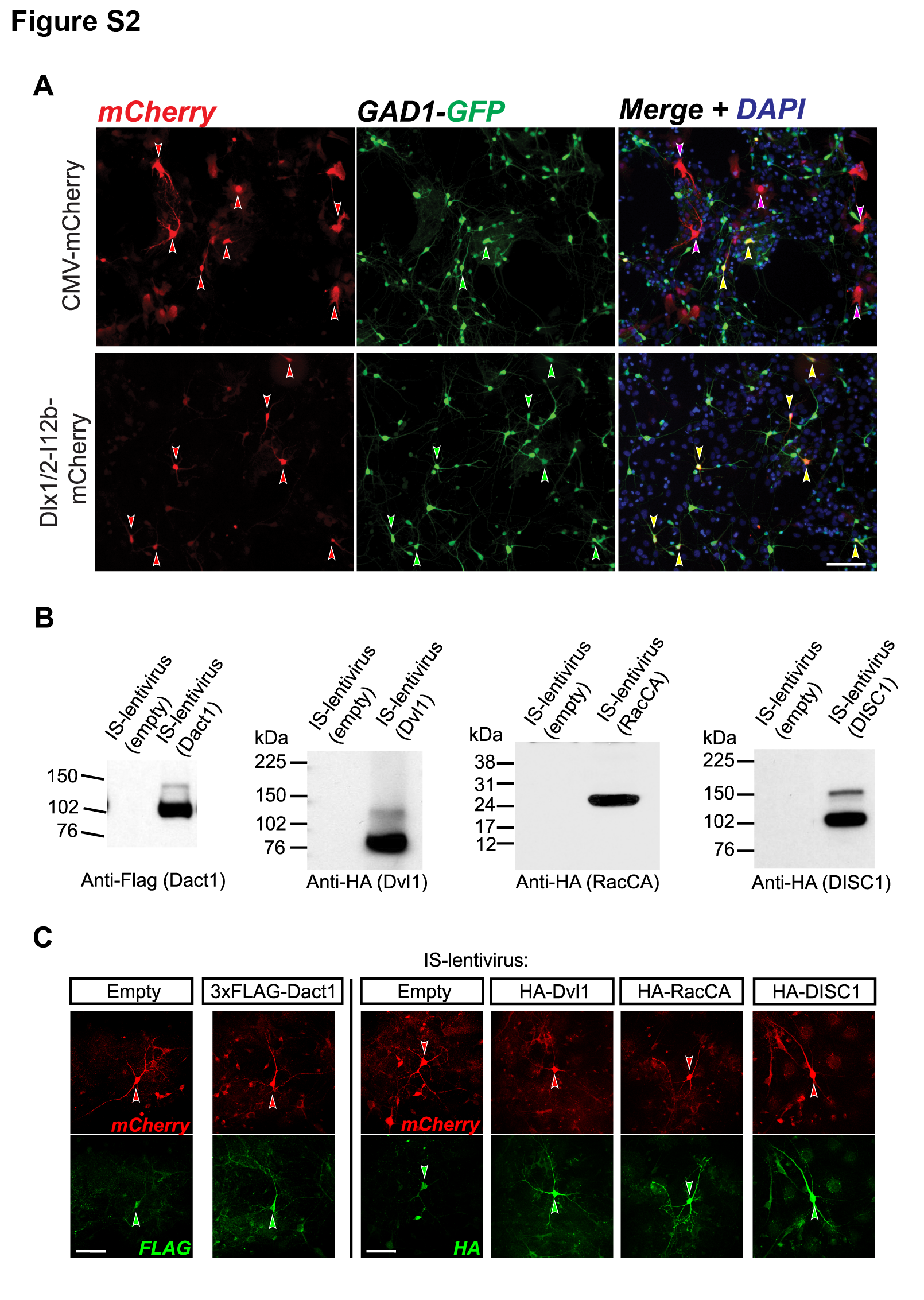

Supplement: Figure S2 — Interneuron specific (IS)-lentivirus drives specific expression in GABAergic interneurons. A Neuronal cultures prepared from postnatal day 0 cortices from GAD1-GFP mice were infected with either a lentiviral construct containing a CMV promoter (top panel) or a Dlx1/2-I12b interneuron specific enhancer (bottom panel) driving mCherry expression. mCherry driven by the CMV promoter containing lentivirus labels some GFP + interneurons (yellow arrowheads, top panel) plus many non-GFP + cells (magenta arrowheads, top panel). mCherry driven by the interneuron specific enhancer containing lentivirus labels only GFP + interneurons (yellow arrowheads, bottom panel). B Human Embryonic Kidney 293T cells were transfected with IS-lentiviral constructs, collected at 3 days post-transfection, and lysates prepared and immunoblotted to confirm specific recombinant protein expression by the constructs used for synapse phenotype rescue experiments. C Neuronal cultures prepared from P0 cortices from wild type mice were infected with IS-lentiviral constructs at DIV1, fixed at DIV7, and stained with either FLAG or HA antibody to confirm recombinant protein expression levels. Scale bars = 100 µm. (TIF) [file pone.0067679.s002.tif]

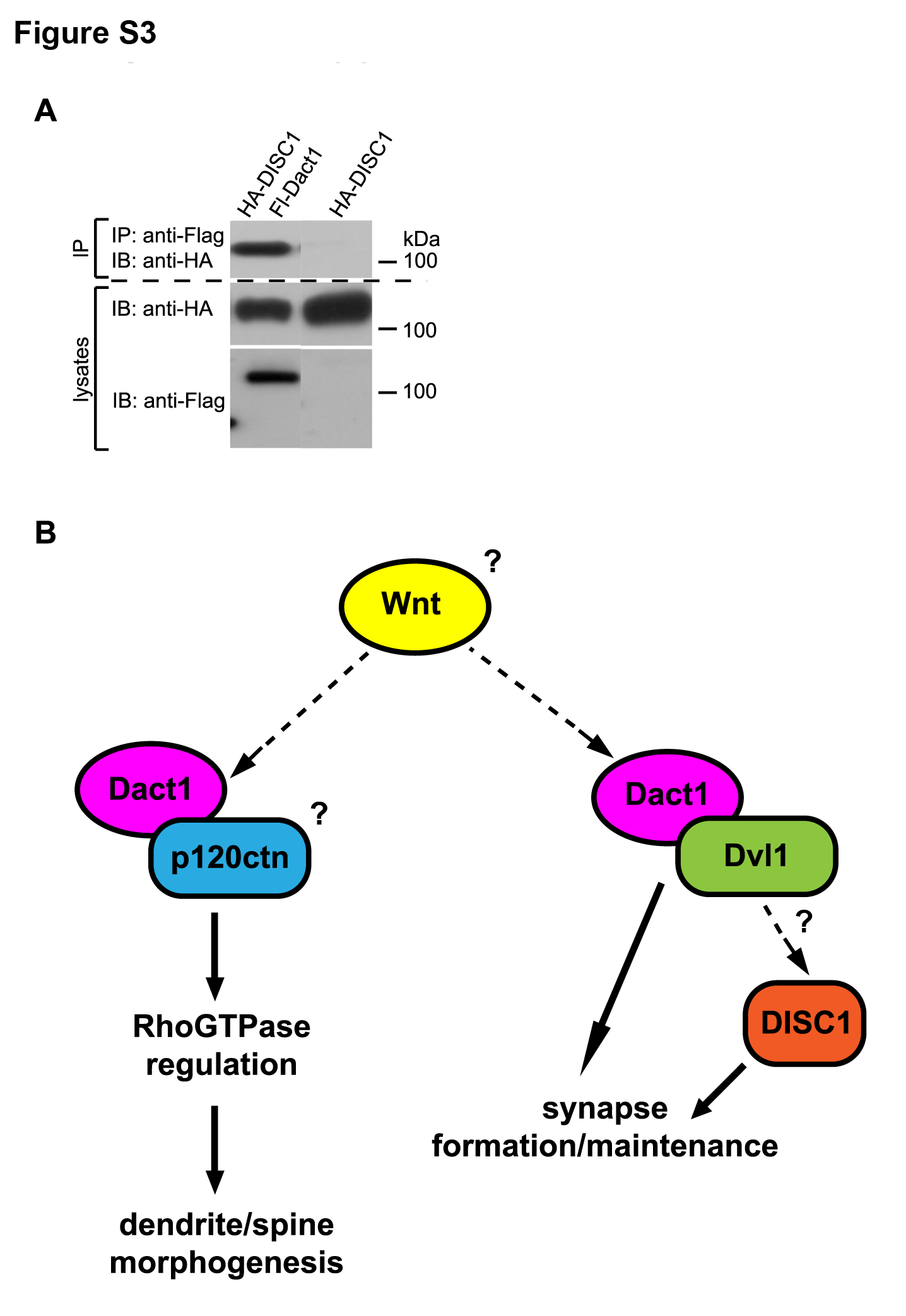

Supplement: Figure S3 — Model reflecting distinct roles for Dact1 in maturing neurons. A Dact1 forms a complex with Disrupted in Schizophrenia-1 (DISC1) when co-expressed in an immortalized human cell line. FLAG-tagged murine Dact1 or HA-tagged murine DISC1 were recombinantly expressed in HEK293T cells, protein complexes immunoprecipitated (IP) with anti-FLAG agarose beads, and associated proteins detected by immunoblot (IB) with anti-HA antibody. B Left: Dact1 promotes actin and other cytoskeletal rearrangements necessary for dendrite and spine formation through a Rac-dependent mechanism that may also involve p120-catenin. Right: Within the postsynaptic compartment Dact1 acts with Dvl1 and possibly with DISC1 (dashed arrow) in synapse formation. Center: Intercellular Wnt ligands and their transmembrane receptor complexes may operate upstream of one or both of these pathways. (TIF) [file pone.0067679.s003.tif]
